# Editorial: Remote assessment, measurement, and delivery in sport, physical activity and health

**DOI:** 10.3389/fspor.2025.1584714

**Published:** 2025-03-14

**Authors:** Daniel J. Peart, Michael Graham, David E. Lunn, Naomi Burn, John D. Franklin, John B. Arnold

**Affiliations:** ^1^ Department of Sport, Exercise and Rehabilitation, Northumbria University, Newcastle upon Tyne, United Kingdom; ^2^ School of Health and Life Sciences, Teesside University, Middlesbrough, United Kingdom; ^3^ Carnegie School of Sport, Leeds Beckett University, Leeds, United Kingdom; ^4^ Alliance for Research in Exercise, Nutrition and Activity (ARENA), University of South Australia, Adelaide, SA, Australia

**Keywords:** remote assessment, measurement, sport, physical activity, health

**Editorial on the Research Topic**
Remote assessment, measurement, and delivery in sport, physical activity and health

## Introduction

Technology has had an increasing influence on our lives for some time, and this was accelerated by the enforced remote way of working five years ago. The first paper in this special topic from Chahin-Inostroza et al. articulates some of the challenges faced during this time as countries across the world enforced distancing measures and remote working to control the spread of COVID-19. Sport was not exempt, and both national and global events were put on standby. However, this not only affected elite athletes, but amateur athletes too had fewer opportunities to socialise with peers and receive feedback and advice. In their cross-section of amateur Chilean athletes, Chahin-Inostroza et al. report how the use of some technologies and training software changed during periods of quarantine, and remained elevated when quarantine ended.

The increase in remote technology acceptance and usage in sport, health and exercise could present an opportunity to move interventions and data collection out of the laboratory or any other fixed location to improve accessibility. However, researchers, practitioners and participants need to be confident that the data collected, processed and analysed in this way is reliable, valid and trustworthy, and that these aspects are not sacrificed as we strive to harness the opportunities and convenience offered by such technology. Therefore, it is important that there is an evolving evidence base as the technology itself evolves.

The goal of this collection was to share evidence regarding remote data collection and intervention in sport, health and exercise sciences to support academics, practitioners and teachers to make evidence informed decisions about the implementation of such technology.

This editorial summarises three areas of interest that are informed by the papers within this special edition.

## Validity of information integration based on subjective and physiological data from a real sports condition: application to the judgment of fatigue in sport

The measurement and assessment of fatigue in both sporting and health care contexts remains challenging (1, 2) however, researchers, practitioners and athletes are becoming increasingly aware of the impact of fatigue on performance and injury risk (3). If researchers can develop a valid and reliable method to effectively measure this construct remotely this could have real life application for developing training sessions with athletes.

The paper by Legall et al. highlights that current subjective measures of fatigue do not allow for the investigation into the possible cognitive processes that may be involved in how an individual generates and perceives fatigue. This paper attempts to explore this by assessing if exercise duration and intensity are used to create a judgement of fatigue. In this study 20 healthy, experienced participants conducted a laboratory session where they provided subjective self-reported fatigue scores based on six different cycling scenarios (15 or 30 min at 30, 50 or 70% maximum intensity). They then undertook each of the six scenarios in a sports hall to see if the perceived fatigue score correlated with the actual fatigue score, as well as collecting objective measures of fatigue. This study shows that all three measures of fatigue correlated with each other, which provides evidence of a correlation between physiological and psychological measures of fatigue. Legall et al. argue that this could be used as a judgement indicator that may allow for more precise and individualised training programmes, although they highlighted that athletes may underestimate their fatigue levels at higher workloads.

## Quantifying lumbar sagittal plane kinematics using a wrist-worn inertial measurement unit

Measuring human movement is important in many different contexts including health, sport and physical activity. Until recently, accurate measurement of human movement has been confined to well controlled laboratory environments. This limits the environments where data is collected and limits the ecological validity of many measurements. However, recent advancements in remote monitoring have enabled human movement measurement outside the laboratory. Examples of remote monitoring in the context of human movement include markerless motion capture (4) and wearable sensors (5).

Wearable sensors, such as inertial measurement units (IMUs), are often small devices attached to the human body and often consist of gyroscopes and accelerometers allowing for a range of biomechanical variables to be calculated. However, the reliability and validity of these devices is still being assessed (6), particularly when movement data can only be measured using devices worn on the area of interest.

To understand the transferability of information from lumbar worn sensors to wrist worn sensors, the paper by Liew et al. in this special issue tested the feasibility of using a wrist-worn IMU to infer lumbar sagittal plane kinematics as a substitute for a lumbar-worn IMU. Eighteen healthy participants performed spinal flexion and extension movements while wearing IMUs on the wrist and lumbar spine. The results showed that flexion range of motion (RoM) was the only variable with a statistically significant difference between sensor locations, with a mean difference of 4.54° (95% CI = 1.82°–7.27°). However, the maximal difference across all outcomes was less than 8°, suggesting that wrist-worn IMUs may provide a practical solution for remote lumbar mobility monitoring, potentially leading to more ergonomically acceptable methods of collecting lumbar spine data. Despite this potential, challenges remain. The accuracy of wrist-worn IMUs may be influenced by individual anthropometric differences and movement speeds. Further research is needed to validate this method across different populations and movement conditions. Nonetheless, wrist-worn IMUs may present an alternative approach for remote lumbar mobility assessment, particularly in rehabilitation contexts where self-monitoring is essential.

## Promoting physical activity and a healthy active lifestyle in community-dwelling older adults: a design thinking approach for the development of a mobile health application

Mobile health (mHealth) applications have become an integral part of modern healthcare, leveraging the ubiquity of smartphones and wearable devices to improve health outcomes, and place a growing emphasis on more accessible, personalised healthcare. These applications encompass a broad range of functions, from monitoring chronic conditions and offering mental health support to providing medication reminders and tracking physical activity (7).

The paper from Daniels et al. in this issue focusses on the public health challenge of engaging older adults (>65 years old) in PA and provides a qualitative synthesis of barriers and facilitators to PA in older adults. Outputs from one-to-one interviews (phase 1) directly informed the co-creation of an mHealth app prototype (phase 2) to promote an active lifestyle to community-dwelling older adults. The authors employ co-creation sessions with older adults and topic experts, underpinned by design thinking methodology (8). The co-produced mHealth prototype was then tested in a population of community-dwelling older adults (phase 3). This study highlights the diverse perceptions of PA engagement in this population, with key considerations for both their own co-created mHealth app and future studies interested in the development of remote PA promotion in older adults.

Challenges persist regarding data privacy and app efficacy (9), along with the need for further evaluation, particularly among those with limited digital access. As mHealth applications continue to develop, it holds the potential to transform how individuals manage their health and interact with healthcare systems globally.

## Summary

Technology in sport and health has accelerated the inclusion of remote data collection and mobile applications. Current research reports on the increased use of technology in this way, the validity and feasibility of these measures, and the development of mobile health apps for promoting physical activity. Despite promising advancements, challenges like data reliability, privacy, and accessibility remain.
